# Incidence and Associated Factors for Chronic Pain After ICU Discharge: A Prospective Study

**DOI:** 10.1111/nicc.70715

**Published:** 2026-09-30

**Authors:** Jinyu Yin, Tianhao Yu, Ruirui Shen, Xiangping Chen, Yinghua Qian, Yuewen Lao, Xiaoyan Gong

**Affiliations:** ^1^ Nursing Department, Sir Run Run Shaw Hospital Zhejiang University School of Medicine Hangzhou Zhejiang China; ^2^ Nursing Department Zhejiang Provincial People's Hospital Hangzhou Zhejiang China

**Keywords:** associated factors, chronic pain, critical care, post‐intensive care syndrome

## Abstract

**Background:**

Survivors of critical illness are at high risk for developing chronic pain. However, evidence regarding new‐onset chronic pain in this specific population remains limited in China.

**Aims:**

This study aimed to investigate the incidence of chronic pain among Chinese intensive care unit (ICU) survivors at 3 months after discharge and to identify associated factors.

**Study Design:**

This was a prospective study. The participants were adult ICU survivors from a mixed ICU of a tertiary hospital in Zhejiang, China. The Brief Pain Inventory (BPI) was used to assess pain at 3 months after ICU discharge. Multivariable binary logistic regression was performed to identify factors independently associated with chronic pain.

**Results:**

Among 328 ICU survivors, 109 (33.2%) reported new‐onset chronic pain, including 64 of 109 (58.7%) with mild pain (BPI average pain score 1–3), 42 of 109 (38.5%) with moderate pain (BPI average pain score 4–6) and 3 of 109 (2.8%) with severe pain (BPI average pain score 7–10). The shoulder was the most frequently reported pain site (46 of 109, 42.2%), and normal work was the daily domain most affected by pain. Multivariable analysis showed that pre‐existing chronic pain (OR = 4.77, 95% CI: 2.02–11.30, *p* < 0.001), days with mechanical ventilation (OR = 1.27, 95% CI: 1.17–1.37, *p* < 0.001), and days with hyperinflammation (OR = 1.34, 95% CI: 1.18–1.53, *p* < 0.001) were independently associated with the development of new‐onset chronic pain. Among 286 patients without pre‐existing chronic pain, 79 (27.6%) developed new‐onset chronic pain; mechanical ventilation duration and hyperinflammation remained independently associated with this outcome.

**Conclusions:**

Approximately one‐third of ICU survivors experience chronic pain, with 41.3% reporting moderate‐to‐severe pain that interfered with daily functioning. These findings highlight the importance of systematic pain assessment and targeted follow‐up after ICU discharge.

**Relevance to Clinical Practice:**

Post‐ICU follow‐up should include systematic assessment of chronic pain and pain‐related functional impairment alongside other PICS domains, including psychological distress, cognitive dysfunction and physical impairments, to ensure holistic, patient‐centred recovery after critical illness.

## Impact Statements

- What is known about the topic
  ○ Chronic pain is a significant issue among ICU survivors and may be associated with mental health disorders such as anxiety and depression, as well as reduced health‐related quality of life.
  ○ Only a limited number of studies have investigated the incidence and associated factors of chronic pain in this population.
- What this paper adds
  ○ In this study, 33.2% of ICU survivors reported new‐onset chronic pain, with 41.3% experiencing moderate‐to‐severe pain.
  ○ Pre‐existing chronic pain, prolonged mechanical ventilation and sustained hyperinflammation were associated with the development of chronic pain.

## 1 Introduction

Advances in life‐saving interventions have led to a growing number of critically ill patients surviving intensive care unit (ICU) admission [1]. However, many survivors experience persistent physical, psychological and cognitive sequelae, collectively termed post‐intensive care syndrome (PICS) [2]. Chronic pain is an under‐reported feature of PICS [3], defined as pain persisting for more than 3 months [4].

Pain is one of the most prevalent and distressing experiences during an ICU stay. Patients are exposed to a wide array of nociceptive stimuli, including underlying disease processes, surgical trauma, invasive procedures and routine nursing care such as repositioning [5, 6, 7]. Despite its high incidence, acute pain is often undertreated or inadequately managed in this setting [8]. The persistence of acute pain in the ICU may be driven by central sensitisation, ongoing systemic inflammation and maladaptive neuroplastic changes within pain‐processing pathways, collectively promoting the chronification of pain following critical illness [9, 10, 11]. This process may be further exacerbated by clinically relevant factors, including high acute pain intensity, prolonged mechanical ventilation and psychological distress [3, 12, 13]. These mechanisms and associated factors underscore the need to understand the epidemiology and impact of chronic pain in this population.

The clinical significance of chronic pain after ICU discharge is substantial. A systematic review reported that the incidence of chronic pain among ICU survivors ranges from 28% to 77%, with joints such as the shoulder and ankle being commonly affected [14]. Bourdiol et al. found that 47.7% of ICU survivors experienced chronic pain at 3‐month follow‐up, yet only 11.3% sought help from a pain specialist [12]. Archer et al. reported that 36% of survivors developed moderate‐to‐severe pain with fear of movement at 2‐year follow‐up [15]. Beyond physical discomfort, chronic pain substantially impairs functional status, activities of daily living and community reintegration. It is also associated with heightened psychological distress and reduced quality of life [16, 17, 18, 19].

Currently, over 2 million patients are admitted to the ICU in China annually [20]. However, Chinese scholars have primarily focused on psychological or cognitive impairments among ICU survivors [21, 22], with limited attention given to chronic pain in this population. Elucidating the clinical characteristics of chronic pain in ICU survivors may facilitate early identification of high‐risk patients, inform the development of targeted interventions and advance post‐ICU rehabilitation research [23, 24].

This study aimed to estimate the incidence of new‐onset chronic pain at 3 months following critical illness and to characterise its severity, clinical features and functional impact. Furthermore, we investigated associated factors for chronic pain development.

## 2 Design and Methods

### 2.1 Setting and Sample

We conducted a prospective study of adult patients admitted to the mixed ICU of a tertiary hospital in Zhejiang, China from December 2024 to March 2025. Patients were eligible for inclusion if they met the following criteria: (1) age ≥ 18 years; (2) ICU length of stay > 48 h; and (3) first‐time ICU admission. Patients were excluded if they had documented cognitive impairment, terminal illness or were transferred to another hospital's ICU. All eligible patients admitted during the study period were invited to participate.

No formal a priori sample size calculation was performed. Instead, consecutive sampling was used to enrol all eligible patients.

On the day of ICU discharge, we informed eligible patients about the study objectives and invited them to participate. After written informed consent was obtained, we retrieved participants' clinical data from electronic medical records and additionally inquired about pre‐existing chronic pain. Information on chronic pain was collected by telephone at 90 days following ICU discharge. Only patients with complete follow‐up data at the 90‐day time point were included in the final analysis.

### 2.2 Data Collection Tools and Methods

#### 2.2.1 Pain

Pain intensity, pain interference and pain location were measured using the Brief Pain Inventory (BPI) [25]. At the 3‐month follow‐up after ICU discharge, patients were first asked a screening question adapted from the first item of the Chinese version of the BPI: ‘Are you experiencing any ongoing pain since discharge from the ICU (only new pain that you did not have before your ICU admission)?’ (yes/no). Patients who answered ‘yes’ were classified as having new‐onset chronic pain and subsequently administered the full BPI. Participants with pre‐existing chronic pain could still be classified as having new‐onset chronic pain if they reported a distinct new pain that had not been present before ICU admission. Pain severity was assessed across four dimensions (worst, least, average and current pain) using an 11‐point numeric rating scale (NRS) ranging from 0 (‘no pain’) to 10 (‘pain as bad as you can imagine’). Based on the average pain score, pain severity was categorised as mild (1–3), moderate (4–6) and severe (7–10) pain [13]. Pain interference was assessed across seven domains: general activity, mood, walking ability, normal work, relationship with other people, sleep and enjoyment of life. Pain location was indicated by the patient marking the affected areas on a body map. The Cronbach's α coefficients for pain intensity and pain interference in the Chinese version of the BPI were 0.894 and 0.915, respectively [26].

#### 2.2.2 Clinical Data

Candidate predictor variables were identified based on a comprehensive literature review and clinical expertise [12, 17, 27]. The following data were extracted from the participants' electronic medical records: age, biological sex, level of education, occupation, body mass index (BMI), smoking, diabetes, surgery, Acute Physiology and Chronic Health Evaluation II (APACHE II) score, diagnosis in ICU, number of days with hyperinflammation (C‐reactive protein > 100 mg/L), number of days with mechanical ventilation, special treatments (including continuous renal replacement therapy [CRRT], extracorporeal membrane oxygenation [ECMO]) and prone position. Sepsis was diagnosed according to the Sepsis‐3 criteria [28]. Opioid use was defined as the administration of at least one dose of any opioid analgesic (including morphine, fentanyl, oxycodone, hydromorphone, etc.) during the patient's entire ICU stay. Pre‐existing chronic pain was assessed via patient self‐report. Participants were asked as follows: ‘Did you have any pain that persisted for more than 3 months before your admission to the ICU?’ (yes/no).

#### 2.2.3 Data Analysis

Based on their responses to the BPI, patients were dichotomised into two groups: those who answered ‘yes’ to the first BPI question were assigned to the new‐onset chronic pain group, whereas those who answered ‘no’ were assigned to the no new‐onset chronic pain group. This binary classification (new‐onset chronic pain vs. no new‐onset chronic pain) served as the dependent variable in subsequent univariate and multivariate logistic regression analyses. Statistical analyses were performed using SPSS Version 26.0 (IBM Corp., Armonk, NY, USA). The normality of continuous variables was tested using the Shapiro–Wilk test. Normally distributed continuous variables were presented as mean ± standard deviation (SD), whereas non‐normally distributed continuous variables were presented as median (interquartile range [IQR]). Categorical variables were expressed as frequencies and percentages. Continuous variables were compared using two‐sided Student's *t*‐test or the Mann–Whitney *U* test. Categorical variables were analysed using the chi‐square test or Fisher's exact test. Univariate analysis was performed on all candidate predictor variables, and those with a *p* value < 0.05 were entered into the multivariate model. A forward stepwise binary logistic regression was then performed to identify independent associated factors for new‐onset chronic pain. For all analyses, a *p* value < 0.05 was considered statistically significant. A total of 109 outcome events (new‐onset chronic pain at 3 months post‐ICU) were observed. Seven variables with *p* < 0.05 in the univariate analyses were entered into the multivariable modelling process. With 109 outcome events, this corresponded to approximately 15.6 events per candidate variable, which exceeds the minimum threshold of 10 recommended by Peduzzi et al. [29].

### 2.3 Ethics Statement

This study was approved by the Ethics Committee of the participating hospital (Approval No. 2024‐2534‐01) (Approval Date: 07 October 2024). Participation was voluntary, and all participants were informed of their right to withdraw from the study at any time without any negative consequences. Written informed consent was obtained from all participants or their legal surrogates prior to enrolment. To ensure data protection and participant confidentiality, all collected data were anonymised and de‐identified prior to analysis. Data were stored on a password‐protected secure server accessible only to the research team.

## 3 Results

### 3.1 Baseline Demographic and Clinical Data

A total of 433 patients were screened for eligibility. After exclusions and losses to follow‐up, 328 patients who completed the 90‐day follow‐up assessment were included in the final analysis (Figure 1).

**FIGURE 1:**
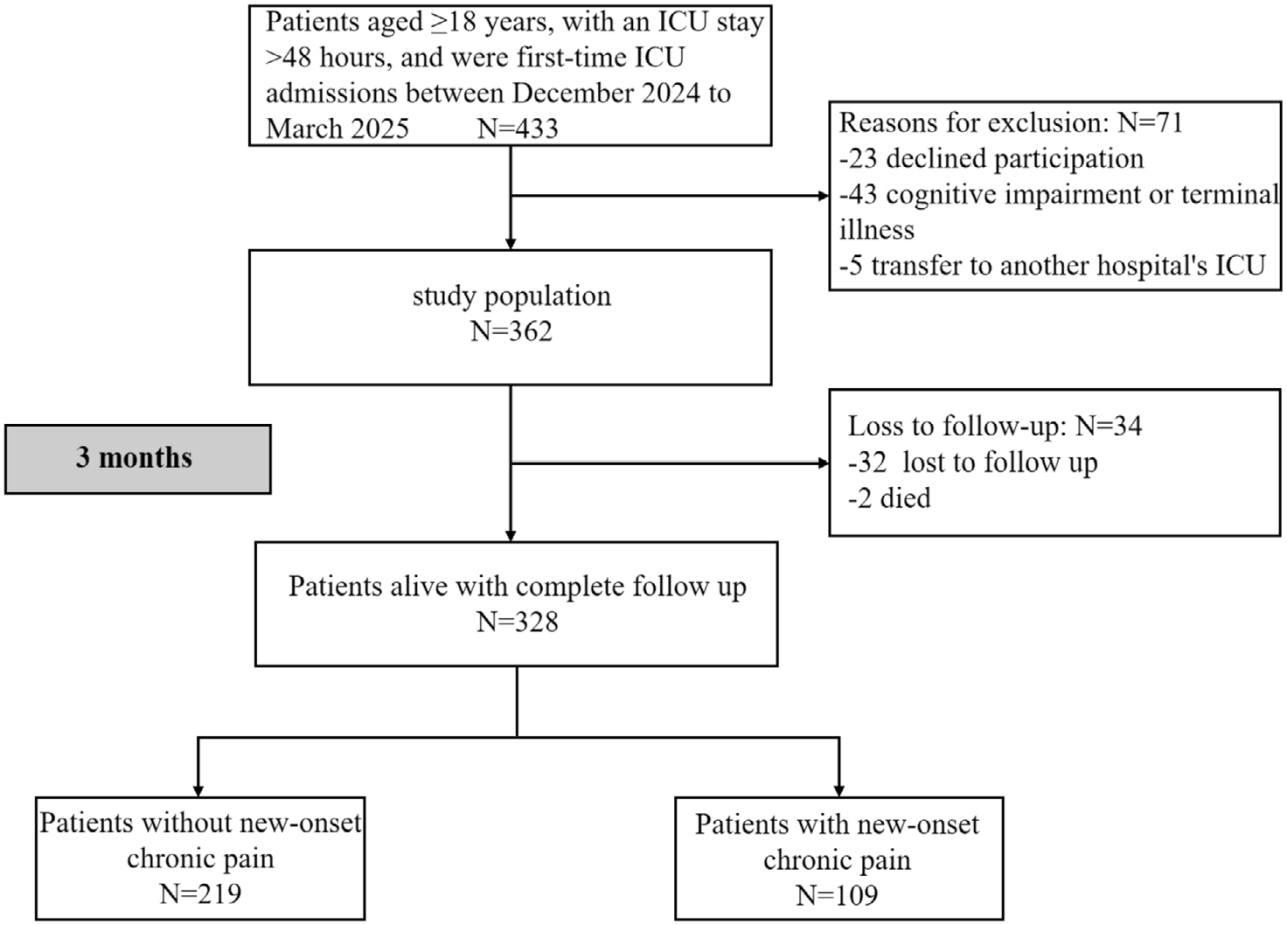
Flowchart of patient inclusion. ICU, intensive care unit; *N*, number of participants.

Among the included patients, 210 (64%) were male, with a median age of 62 (IQR, 51–72). Patients were primarily hospitalised for circulation system diseases (38.4%), digestive system diseases (25%) and respiratory system diseases (12.8%). A total of 42 (12.8%) reported pre‐existing chronic pain, and 167 (50.9%) received opioid medications during their ICU stay. 188 (57.3%) patients underwent surgical procedures during their ICU stay. The median ICU length of stay was 5 days (IQR, 3–8), mechanical ventilation duration was 2 days (IQR, 1–5), and hyperinflammation duration was 1 day (IQR, 0–3) (Table 1). Furthermore, among the 249 patients who received mechanical ventilation, the median duration was 3 days (IQR, 2–7); among the 171 patients with hyperinflammation, the median duration was 3 days (IQR, 2–4) (Table S1).

**TABLE 1:** Baseline demographic and clinical data.

Characteristic	No new‐onset chronic pain, *N* = 219	New‐onset chronic pain, *N* = 109	*p*
Biological sex, *n* (%)			
Male	143 (65.3)	67 (61.5)	0.496
Female	76 (34.7)	42 (38.5)	
Level of education, *n* (%)			
Primary school or below	103 (47.0)	45 (41.3)	0.715
Junior high school	54 (24.7)	32 (29.4)	
Senior high school	30 (13.7)	14 (12.8)	
College or above	32 (14.6)	18 (16.5)	
Occupation, *n* (%)			
Unemployed/retired	100 (45.7)	48 (44.0)	0.873
Farmer	41 (18.7)	21 (19.3)	
Worker	35 (16.0)	21 (19.3)	
Company employee	43 (19.6)	19 (17.4)	
Smoking, *n* (%)	45 (20.6)	29 (26.6)	0.216
Special treatment (CRRT/ECMO), *n* (%)	25 (11.4)	21 (19.3)	0.054
Prone position, *n* (%)	10 (4.6)	8 (7.3)	0.299
Sepsis, *n* (%)	8 (3.7)	10 (9.2)	0.039
Diabetes, *n* (%)	44 (20.1)	28 (25.7)	0.249
Diagnosis in ICU, *n* (%)			
Respiratory system diseases	22 (10.1)	20 (18.4)	0.143
Circulation system disease	93 (42.5)	33 (30.3)	
Digestive system diseases	55 (25.1)	27 (24.8)	
Nervous system diseases	9 (4.1)	8 (7.3)	
Trauma	11 (5.0)	6 (5.5)	
Other	29 (13.2)	15 (13.8)	
Pre‐existing chronic pain, *n* (%)	12 (5.5)	30 (27.5)	< 0.001
Mechanical ventilator treatment, *n* (%)	146 (66.7)	83 (76.2)	0.078
Opioid use, *n* (%)	92 (42.0)	75 (68.8)	< 0.001
Surgery, *n* (%)	132 (60.3)	56 (51.4)	0.125
Age, year, median (IQR)	61 (51–71)	63 (54–74)	0.137
BMI, kg/m^2^, median (IQR)	22.6 (20.8–25.2)	22.7 (21.1–24.8)	0.751
APACHE II, score, median (IQR)	15 (11–20)	18 (14–23)	< 0.001
Number of days with, days, median (IQR)			
ICU length of stay	4 (3–5.5)	8 (5–14)	< 0.001
Mechanical ventilation	2 (0–3)	6 (3–10)	< 0.001
Hyperinflammation	0 (0–2)	3 (0–5)	< 0.001

*Note:* The normality of continuous variables was assessed using the Shapiro–Wilk test. As all continuous variables were not normally distributed (*p* < 0.05), they are presented as median (IQR) and compared using the Mann–Whitney *U* test. All categorical variables were compared using Pearson's chi‐squared test, as the expected frequencies in all contingency tables were ≥ 5. A *p* value < 0.05 was considered statistically significant.

Abbreviations: APACHE II, Acute Physiology and Chronic Health Evaluation II; BMI, body mass index; IQR, interquartile range.

### 3.2 Incidence, Location and Characteristics of New‐Onset Chronic Pain

At 3 months after ICU discharge, 109 of 328 (33.2%) patients experienced new‐onset chronic pain, including 64 of 109 (58.7%) with mild pain, 42 of 109 (38.5%) with moderate pain and 3 of 109 (2.8%) with severe pain. 59 of 109 patients (54.1%) reported two or more sites of pain. The most commonly reported pain locations were the shoulders (46/109, 42.2%), followed by the back (27/109, 24.8%), legs (24/109, 22.0%), chest (21/109, 19.3%) and abdomen (10/109, 9.2%). New‐onset chronic pain significantly interfered with various aspects of daily life, with the greatest interference on patients' normal work, followed by general activity, sleep, mood, walking ability, relationship with other people and enjoyment of life (Figure 2). Sub‐sample analysis comparing patients with pre‐existing chronic pain (*n* = 30) and those without pre‐existing chronic pain who developed new‐onset chronic pain after ICU discharge (*n* = 79) revealed no significant difference in pain intensity between the two groups (*p* = 0.378). For pain interference, patients without pre‐existing chronic pain reported significantly higher interference in general activity (*p* = 0.032) compared to those with pre‐existing pain. No significant differences were observed in other interference domains (*p* > 0.05 for all). Detailed results are presented in Table 2.

**FIGURE 2:**
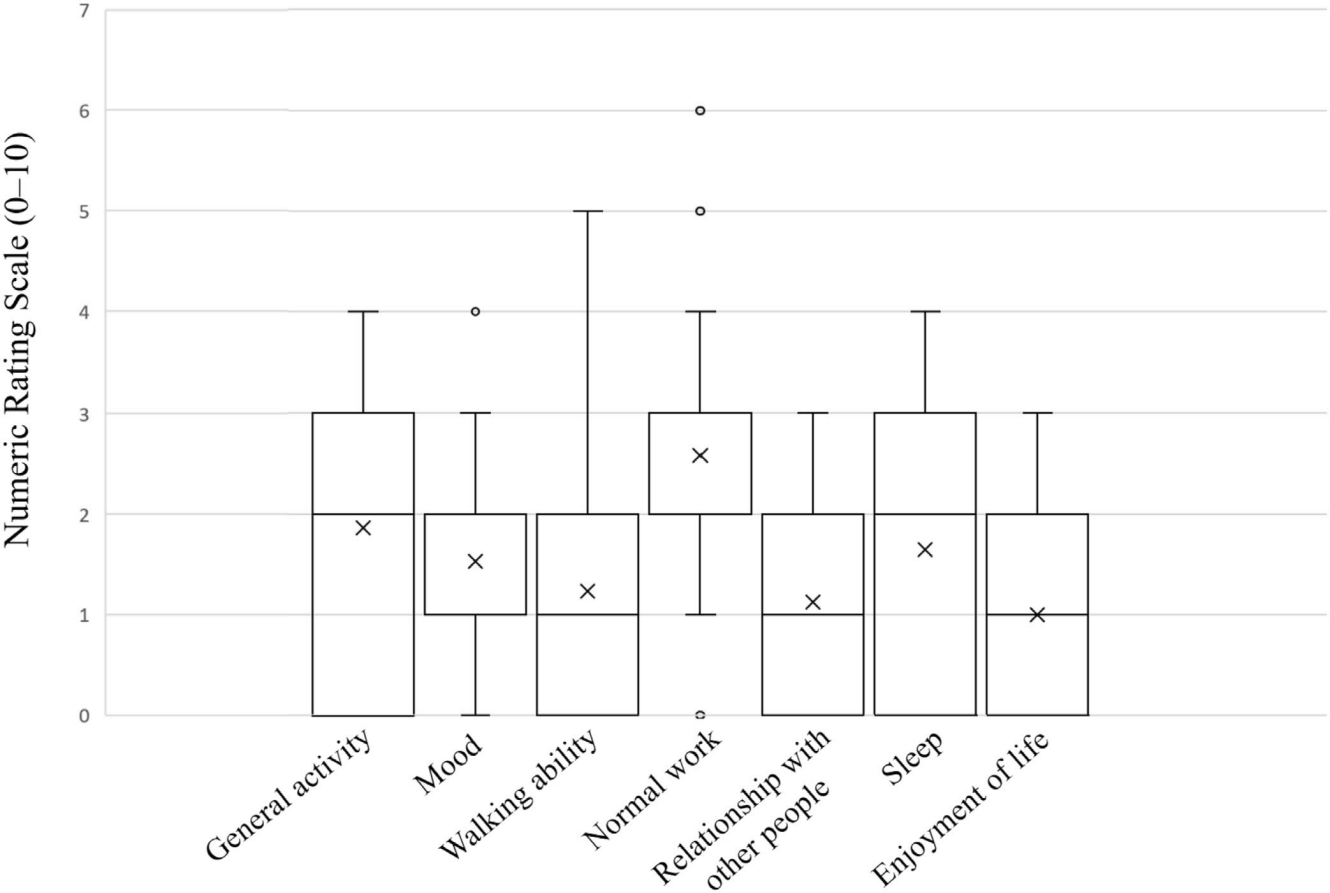
Distribution of pain interference scores across seven domains among ICU survivors with new‐onset chronic pain at 3‐month follow‐up (*N* = 109). Data are presented as box plots: The central line represents the median, box boundaries represent the interquartile range (IQR, 25th to 75th percentiles), the ‘×’ symbol indicates the mean, and whiskers extend to the minimum and maximum values within 1.5 × IQR from the box edges. Points beyond the whiskers represent outliers. The *Y*‐axis is displayed from 0 to 7 (rather than the full 0–10 NRS range) to improve visualisation of the data distribution. BPI, Brief Pain Inventory; ICU, intensive care unit; IQR, interquartile range.

### 3.3 Associated Factors for New‐Onset Chronic Pain

Variables with statistical significance in univariate analyses were entered into the multivariate analysis. These variables included pre‐existing chronic pain, APACHE II score, opioid use, sepsis, length of ICU stay and number of days with mechanical ventilation and hyperinflammation (Table 1).

In the multivariable analysis, the factors independently associated with new‐onset chronic pain were pre‐existing chronic pain (OR = 4.77, 95% CI: 2.02–11.30, *p* < 0.001), days with mechanical ventilation (OR = 1.27, 95% CI: 1.17–1.37, *p* < 0.001) and days with hyperinflammation (OR = 1.34, 95% CI: 1.18–1.53, *p* < 0.001). The independent associated factors for chronic pain are shown in Table 2.

**TABLE 2:** Multivariable logistic regression analysis of associated factors for new‐onset chronic pain.

Associated factors	OR	95% CI	*p*
Pre‐existing chronic pain			
No (reference)	1.00 (reference)	—	—
Yes	4.77	2.02–11.30	< 0.001
Days with mechanical ventilation	1.27	1.17–1.37	< 0.001
Days with hyperinflammation	1.34	1.18–1.53	< 0.001

*Note:* This table presents the results of multivariable logistic regression analysis identifying independent factors for new‐onset chronic pain in ICU survivors. Variables with a *p* value < 0.05 in univariate analysis were considered candidate variables and entered into the multivariable model; a forward stepwise selection method was applied to identify independent factors. Data are presented as ORs with 95% CIs and *p* values. A *p* value < 0.05 was considered statistically significant.

Abbreviations: CI, confidence interval; OR, odds ratio.

Subgroup analysis was performed in patients without pre‐existing chronic pain (*n* = 286). In this subgroup, the incidence of new‐onset chronic pain following ICU discharge was 27.6% (79/286). Variables with *p* < 0.05 in univariate analysis (including smoking, sepsis, opioid use, APACHE II score, ICU length of stay and days with mechanical ventilation and hyperinflammation) were entered into the multivariate model. Multivariate logistic regression analysis revealed that days with mechanical ventilation (OR = 1.26, 95% CI: 1.16–1.36, *p* < 0.001) and days with hyperinflammation (OR = 1.38, 95% CI: 1.21–1.59, *p* < 0.001) were independently associated with new‐onset chronic pain (Table 3).

## 4 Discussion

Chronic pain is an important issue for ICU survivors, and there is growing awareness that it reduces quality of life [30]. In our prospective study, we found that nearly one‐third of ICU survivors reported new‐onset chronic pain at 90 days. The intensity of chronic pain is predominantly mild to moderate, involves multiple body locations and significantly interferes with several domains of daily life, including activity, mood, work and sleep. Factors independently associated with the development of new‐onset chronic pain included pre‐existing chronic pain, longer duration of mechanical ventilation and prolonged hyperinflammation during the ICU stay.

### 4.1 Incidence, Location and Characteristics of New‐Onset Chronic Pain

Our study found that the incidence of new‐onset chronic pain among Chinese ICU survivors was 33.2%. Data on the incidence of chronic pain in this population remain limited, with a few studies reporting incidence rates ranging from 49% to 77% at 3 months post‐ICU discharge [4, 16, 31, 32]. This discrepancy may be partly attributable to bias introduced by loss to follow‐up, as some studies reported response rates of approximately 60% [4]. Additionally, compared to participants in Hayhurst's study [16], our cohort had lower APACHE II scores, suggesting less severe illness, which may also contribute to the relatively lower incidence observed in our study. Cultural attitudes may also influence pain perception, reporting and help‐seeking behaviours [33]. A national survey in China found that 24.06% of respondents did not seek medical help, and 36.79% never received any treatment because they considered their pain to be ‘not harmful’ [34]. Such cultural norms, which minimise the seriousness of pain and normalise it as a benign condition, may contribute to an underestimation of the true burden of post‐ICU chronic pain. Nevertheless, our findings indicate a substantial burden of chronic pain among ICU survivors. Given that the number of ICU survivors is expected to increase in the future [35], a 33.2% incidence of new‐onset chronic pain within 3 months after ICU discharge represents a considerable clinical and public health concern. Even among patients without pre‐existing chronic pain, the incidence remained substantial at 27.6%, further supporting the clinical significance of post‐ICU chronic pain. The absolute number of affected individuals is likely to rise accordingly.

One of the most common pain locations identified in our study was the shoulder, nearly twice as common as in another study that found over 25% of patients experienced shoulder pain following ICU admission [36]. Multiple studies have indicated that the shoulder is the most common pain site [37]. It has been reported that 73% of ICU survivors experienced shoulder injuries within 3 months after ICU discharge [38], and these injuries were difficult to recover from without intervention [39]. One contributing factor to shoulder pain may be ICU‐acquired weakness, which can cause decreased muscle tone, leading to loss of shoulder joint stability and resultant pain [40]. Other possible causes include the shoulder being a common site for various invasive equipment (such as central venous catheters, pulmonary artery catheters, dialysis catheters) and improper patient repositioning techniques [4, 41]. One study also identified the abdomen as the most common pain location, which may be attributed to the fact that 86% of its study population consisted of abdominal surgery patients [32]. These findings highlight the importance of tailored pain assessment based on patient characteristics and clinical context. Healthcare providers should prioritise early identification of patients at high risk for chronic pain, implement protective strategies during invasive procedures and strictly adhere to standardised pain management protocols to minimise procedure pain [42, 43]. Early mobilisation during the ICU stay has been proposed as a potential preventive intervention for chronic pain [44]. It may enhance muscle strength, prevent joint contractures and exert a protective effect on areas susceptible to chronic pain [45]. However, its effectiveness in preventing chronic pain remains inconclusive [46], and further high‐quality studies are needed to determine its role in pain prevention. Given that a longer duration of mechanical ventilation (a marker of immobility) was identified as an independent associated factor for chronic pain in our study, early mobilisation may represent a modifiable target for future intervention studies aimed at reducing post‐ICU chronic pain.

Among the seven domains assessed, the highest level of interference was observed in normal work and general activity, whereas enjoyment of life showed the lowest interference. These findings are consistent with those reported by Langerud et al. [4]. Our results suggest that even mild but persistent pain may adversely affect daily functioning. Furthermore, inadequate follow‐up care may exacerbate the long‐term consequences of chronic pain over time [47]. This underscores the need for healthcare professionals to implement systematic follow‐up for ICU survivors, which may confer potential benefits in improving both chronic pain outcomes and daily functioning.

### 4.2 Associated Factors for New‐Onset Chronic Pain

In our study, pre‐existing chronic pain was identified as an independent associated factor for post‐ICU chronic pain, a finding consistent with the results of a study from Germany by Baumbach et al. [48], who similarly reported pre‐existing chronic pain as a predictor of chronic pain following critical illness. Prolonged exposure to pain stimuli during critical illness can induce neuroplastic changes and central sensitisation in ICU survivors, potentially contributing to the development of hyperalgesia and chronic pain [49]. This process may be further amplified in patients with pre‐existing chronic pain conditions. To examine whether the observed associations were consistent among patients without prior chronic pain, we performed a subgroup analysis restricted to these participants. Longer durations of mechanical ventilation and hyperinflammation remained independently associated with new‐onset chronic pain in this subgroup, indicating that these associations were not solely attributable to pre‐existing pain.

A previous study from Norway showed that patients receiving mechanical ventilation lasting more than 12 days are at increased risk of developing chronic pain [4]. Our study similarly identified mechanical ventilation duration as an associated factor for chronic pain. The duration of mechanical ventilation can serve as a marker of disease severity, which may be associated with the development of chronic pain [50]. Moreover, procedures such as tracheal intubation and endotracheal suctioning are common sources of acute pain [43], and inadequate management of acute pain may facilitate its transition to chronic pain. Mechanically ventilated patients may also be exposed to factors associated with ICU‐acquired weakness, including prolonged immobilisation and certain medications, which may contribute to subsequent musculoskeletal dysfunction [51, 52].

Our study revealed that the number of days with hyperinflammation during ICU stay was significantly associated with chronic pain, consistent with the findings of a study from the Netherlands by Koster‐Brouwer et al. [36]. Accumulating evidence suggests that excessive inflammatory responses play a pivotal role in the transition from acute to chronic pain [53]. Inflammation triggers the release of inflammatory mediators, which directly or indirectly stimulate nociceptors, increasing the sensitivity of peripheral nociceptors and lowering the pain threshold. This peripheral sensitisation not only contributes to the onset of acute pain but also promotes its persistence, ultimately facilitating the chronification of pain [54].

Previous studies have identified associated factors for chronic pain across multiple domains, including demographic and clinical characteristics. Baumbach et al. administered the German Pain Questionnaire to patients within 6 months of ICU discharge, and identified age, gender and APACHE II as factors associated with chronic pain [17]. Bourdiol et al. defined chronic pain as NRS ≥ 3 points and found that gender, prone positioning and admission diagnosis were significantly associated with chronic pain [12]. Probert et al. assessed pain in acute respiratory distress syndrome survivors at 6 months post‐ICU discharge using the Short Form‐36 Bodily Pain (SF‐36 BP) scale and reported that chronic pain incidence was associated with BMI, smoking history and opioid use [27]. Martinez et al. conducted face‐to‐face pain assessments using the BPI in COVID‐19 ICU survivors at 9 months post‐discharge. The study reported that intubation during the ICU stay was significantly more prevalent among patients who developed new‐onset chronic pain than among those who did not [13]. In contrast, our study found that only pre‐existing chronic pain, days with mechanical ventilation and days with hyperinflammation were independently associated with chronic pain. These discrepancies may be attributed to variations in assessment tools, timing of pain evaluation, study populations and data collection methods across studies [14, 55, 56]. Consequently, the factors associated with chronic pain remain somewhat inconclusive, and our findings should be interpreted with caution. Nevertheless, despite these limitations, our study offers preliminary evidence regarding factors associated with chronic pain in a Chinese ICU survivor cohort—a population that has received limited research attention in this field. To date, no chronic pain prediction model has been specifically developed or validated for Chinese ICU survivors. Within this context, our findings may serve as a foundational reference for the early identification of high‐risk patients and for informing the design of culturally appropriate, clinically feasible intervention strategies in future research.

### 4.3 Strengths and Limitations

Our study offers several strengths. To the best of our knowledge, this represents one of the few investigations to date that have focused specifically on factors associated with chronic pain among ICU survivors in China, a population that remains largely understudied in this context. The use of a validated instrument (the BPI) and a relatively large sample size (*N* = 328) further strengthens the validity of our findings.

Several limitations should also be acknowledged. First, although patients were instructed to report only new pain since ICU admission that had not been present before, we did not explicitly ask whether they attributed this pain to the ICU stay. Consequently, some chronic pain cases may have developed independently of the ICU episode, potentially introducing misclassification bias. Data on pre‐existing chronic pain were entirely based on patient recall, which is subject to recall bias. Second, we only collected binary data on whether patients received analgesic drugs during their ICU stay, without obtaining complete records of analgesic use before ICU admission or after discharge, nor did we record whether patients visited a doctor specifically for pain management during the follow‐up period. In addition, no continuous dynamic assessment was conducted to verify the adequacy of pain management during the ICU stay. This limits our ability to assess the impact of pain management adequacy on chronic pain outcomes. Third, emotional symptoms after ICU discharge were not investigated, although they may be closely associated with chronic pain. Fourth, this is a single‐centre study and may therefore reflect only regional patient characteristics, limiting the generalisability of our findings to the broader population. Finally, the 90‐day follow‐up period may not fully reflect the long‐term trajectory of pain. Extended observation periods and dedicated assessment of health‐related quality of life would therefore be of significant value in future investigations.

### 4.4 Implications and Recommendations for Practice

Post‐ICU follow‐up should include systematic assessment of chronic pain and pain‐related functional impairment alongside other PICS domains, including psychological distress, cognitive dysfunction and physical impairments, to ensure holistic, patient‐centred recovery after critical illness.

## 5 Conclusion

Chronic pain is a common health problem among ICU survivors. Our findings indicate that pre‐existing chronic pain, prolonged mechanical ventilation and sustained hyperinflammation are independently associated with chronic pain after ICU discharge. These findings support closer pain assessment during post‐ICU follow‐up.

## Author Contributions

**Jinyu Yin:** writing – original draft, resources, project administration, methodology, investigation, formal analysis, data curation, conceptualisation. **Tianhao Yu:** writing – review and editing, visualisation, validation, supervision, methodology, investigation, formal analysis, data curation, conceptualisation. **Ruirui Shen:** writing – review and editing, visualisation, validation, supervision, methodology, investigation, formal analysis, data curation, conceptualisation. **Xiangping Chen:** conceptualisation, methodology, software, validation, formal analysis, investigation, resources, data curation, writing – review and editing, supervision. **Yinghua Qian:** writing – review and editing, visualisation, validation, supervision, methodology, conceptualisation. **Yuewen Lao:** writing – review and editing, validation, supervision, methodology, conceptualisation. **Xiaoyan Gong:** writing – review and editing, validation, supervision, methodology, conceptualisation.

## Funding

This work was supported by Nursing Research Fund of Zhejiang University School of Medicine Sir Run Run Shaw Hospital (grant no. 2026HLKY18) awarded to Jinyu Yin.

## Ethics Statement

This study was approved by the Ethics Committee of Sir Run Run Shaw Hospital, Zhejiang University School of Medicine (approval code: 2024‐2534‐01; approval date: 07‐10‐2024). Written informed consent was obtained from all participants or their legal surrogates prior to enrolment.

## Conflicts of Interest

The authors declare no conflicts of interest.

## Supporting information


**Table S1:** Comparison of mechanical ventilation and hyperinflammation durations between patients with or without new‐onset chronic pain.
**Table S2:** Comparison of pain intensity and interference between patients with or without pre‐existing chronic pain.
**Table S3:** Multivariable logistic regression analysis of factors associated with new‐onset chronic pain among patients without pre‐existing chronic pain.

## Data Availability

The data that support the findings of this study are available from the corresponding author upon reasonable request. The data are not publicly available due to privacy and ethical restrictions, as they contain information that could compromise the confidentiality of research participants.
