# Supplementary material for: Incidence and Associated Factors for Chronic Pain After ICU Discharge: A Prospective Study
Source: Nurs Crit Care. 2026 Sep 30;31(6):e70715. doi: 10.1111/nicc.70715 (PMC13625663; doi:10.1111/nicc.70715)
Supplement: Supplementary file 1 [file NICC-31-0-s001.docx]

Supplementary Table 1 Comparison of mechanical ventilation and hyperinflammation durations between patients with or without new-onset chronic pain

| Characteristic | N | Patients without new-onset chronic pain | Patients with new-onset chronic pain | *P* |
| --- | --- | --- | --- | --- |
| Mechanical ventilation, days, Median (IQR) | 249 | 2(2-3) | 7(4-12) | <0.001 |
| Hyperinflammation, days, Median (IQR) | 171 | 2(2-3) | 4(3-6.5) | <0.001 |

Abbreviations: IQR, interquartile range. Data are presented as median (IQR) and were restricted to patients who actually experienced the corresponding event (excluding patients with 0 days). *P* values were calculated using the Mann-Whitney U test. A *P*-value < 0.05 was considered statistically significant.

Supplementary Table 2 Comparison of pain intensity and interference between patients with or without pre‑existing chronic pain

| Characteristic | Patients without pre-existing chronic pain  N=79 | Patients with pre-existing chronic pain  N=30 | *P* |
| --- | --- | --- | --- |
| Pain intensity, score, median (IQR) | 3(2-4) | 4(2-4) | 0.378 |
| Pain interference, score, median (IQR) |  |  |  |
| general activity | 2(1-3) | 1(0-3) | 0.032 |
| mood | 2(1-2) | 2(0-2) | 0.528 |
| walking ability | 1(0-2) | 1(0-2) | 0.688 |
| normal work | 3(1-4) | 2(2-3) | 0.256 |
| relationships with other people | 1(0-2) | 2(0-2) | 0.134 |
| sleep | 2(0-3) | 2(0-2) | 0.468 |
| enjoyment of life | 1(0-2) | 1(0-2) | 0.253 |

A *P*-value < 0.05 was considered statistically significant.

Supplementary Table 3 Multivariable logistic regression analysis of factors associated with new-onset chronic pain among patients without pre-existing chronic pain

| Associated Factors | OR | 95% CI | *P* value |
| --- | --- | --- | --- |
| Days with mechanical ventilation | 1.26 | 1.16-1.36 | <0.001 |
| Days with hyperinflammation | 1.38 | 1.21-1.59 | <0.001 |

A *P*-value < 0.05 was considered statistically significant.

Abbreviations: OR, odds ratio; CI, confidence interval.
